# Enhancing the photoelectrochemical performance of BiOI-derived BiVO_4_ films by controlled-intensity current electrodeposition

**DOI:** 10.3762/bjnano.16.94

**Published:** 2025-08-07

**Authors:** Huu Phuc Dang, Khanh Quang Nguyen, Nguyen Thi Mai Tho, Tran Le

**Affiliations:** 1 Faculty of Fundamental Science, Industrial University of Ho Chi Minh City, Ho Chi Minh City, Vietnam; 2 Advanced Materials and Applications Research Group (AMA), HUTECH University, 475A Dien Bien Phu Street, Binh Thanh District, Ho Chi Minh City 700000, Vietnam; 3 Faculty of Chemical Engineering, Industrial University of Ho Chi Minh City, Ho Chi Minh City, Vietnam; 4 Faculty of Physics & Engineering Physics, VNUHCM-University of Science, Ho Chi Minh City, Vietnam; 5 Vietnam National University, Ho Chi Minh City, Vietnam

**Keywords:** BiOI, BiVO_4_, electrodeposition, photoelectrochemical water splitting

## Abstract

This study investigates the fabrication of BiVO_4_ photoanodes using a controlled-intensity current electrodeposition method to improve their photoelectrochemical (PEC) performance. The impact of varying the deposition current density and VO(acac)_2_ concentration was systematically analyzed to optimize the crystallinity, surface morphology, and electronic properties of the films. Subsequently, an electrochemical deposition method was developed to facilitate the uniform distribution of V_2_O_5_ among Bi–O–I flakes to homogeneously enhance the conversion reaction. The XRD pattern confirms the monoclinic scheelite BiVO_4_ structure with dominant (121) and (004) peaks. FESEM imaging revealed that the different deposition conditions influenced the surface morphologies of the BiOI and BiVO_4_ films. Photocurrent density measurements showed that BiVO_4_(326) achieved 1.2 mA·cm^−2^ at 1.23 V vs RHE, representing a significant enhancement compared to the other samples. The surface hole injection efficiency was measured to be 47%, whereas the incident photon-to-current efficiency reached a peak of 18.1% at 420 nm. The applied bias photon-to-current efficiency of BiVO_4_(326) was also superior to that of the samples fabricated with lower current density, highlighting the benefits of the optimized electrodeposition conditions for the former.

## Introduction

In the context of the increasing global energy demand, the development of renewable and sustainable energy sources has become a top priority in science and technology [1–2]. Photoelectrochemical (PEC) water-splitting systems hold significant promises for converting abundant solar energy into chemical fuels, such as hydrogen [3–4]. However, their widespread application is still limited by material challenges, including insufficient light absorption, high electron–hole recombination rates, and poor stability under operating conditions [5–6]. Among various semiconductor materials, bismuth vanadate (BiVO_4_) has attracted considerable interest due to its strong visible light absorption, moderate bandgap (≈2.4 eV), high theoretical photocurrent density (≈7.5 mA·cm^−2^), and chemical stability in aqueous environments [7–9]. Nevertheless, BiVO_4_ suffers from intrinsic drawbacks such as low charge carrier mobility, limited conductivity, and rapid recombination of photogenerated charge carriers, which severely restrict its PEC performance [10–12].

Various strategies have been explored to overcome these challenges and optimize the structural, electronic, and surface properties of BiVO_4_ [13–14]. Hydrothermal synthesis has been used to produce highly crystalline BiVO_4_ films with large surface areas; for instance, Yun He et al. [15] reported flower-like BiVO_4_ photoanodes achieving a photocurrent density of 0.81 mA·cm^−2^ at 1.23 V vs RHE. However, the hydrothermal method often requires high temperatures and prolonged reaction times, and offers limited control over film thickness. Alternatively, Liu et al. [16] employed RF sputtering with a single BiVO_4_ target, but the volatility of Bi in a vacuum environment often led to an imbalanced Bi/V ratio, requiring precise regulation of oxygen partial pressure. Gong et al. [17] utilized DC co-sputtering of Bi and V targets to produce BiVO_4_ thin films at high deposition rates; however, this method resulted in irregular grain structures and significant material defects, limiting the PEC performance improvements. Electrodeposition has emerged as a promising low-cost and scalable technique for BiVO_4_ film fabrication, offering better control over film morphology and crystallinity under mild conditions. Kim et al. [18] reported that BiVO_4_ films fabricated via electrodeposition achieved a maximum photocurrent density of 1.4 mA·cm^−2^ at 1.23 V vs RHE. These films exhibited a three-dimensional nanoporous structure that facilitated charge carrier transport; however, their uneven porosity and high charge recombination rates hindered their PEC performance improvement. McDonald and Choi [19] introduced a facile electrodeposition method based on *p*-benzoquinone reduction to fabricate ultrathin BiOI films, which could be thermally converted into porous BiVO_4_ photoanodes. This approach yielded electrodes with enhanced PEC activity, achieving a photocurrent density of approximately 1.25 mA·cm^−2^ at 0.5 V vs RHE in neutral phosphate buffer under AM1.5G illumination. This study highlighted the potential of BiOI-derived BiVO_4_ as a template-guided route for improving water oxidation performance. However, the use of a constant-potential deposition technique presents limitations in controlling the film thickness, morphology, and uniformity, which are crucial for consistent BiVO_4_ performance after conversion. Variability in the BiOI film quality remains a significant challenge, affecting the reproducibility and optimization of the final photoanodes.

Building on these limitations, in this study, we introduce a novel controlled-intensity current electrodeposition method to precisely tailor the deposition conditions of BiOI and subsequently optimize its conversion to BiVO_4_. By systematically adjusting the deposition current density and vanadium precursor concentration, we achieved fine control over the crystallinity, grain size, porosity, and optical properties of the resulting films. This level of tunability leads to substantial improvements in PEC performance. Our method offers a higher degree of control over both the intermediate BiOI layer and the final BiVO_4_ structure, thereby enabling enhanced charge separation and surface reaction kinetics. Furthermore, this approach provides a deeper understanding of the relationship between the synthesis parameters and PEC activity, while presenting a scalable and reproducible route for fabricating high-performance BiVO_4_ photoanodes.

## Experimental

### Material

Bismuth nitrate pentahydrate (Bi(NO_3_)_3_·5H_2_O, 99.9%, Sigma-Aldrich) and vanadyl acetylacetonate (VO(acac)_2_, 98%, Sigma-Aldrich) were used as Bi and V sources, respectively. Potassium iodide (KI, 99.5%, Sigma-Aldrich) was used for the initial deposition of BiOI. *p*-Benzoquinone (C_6_H_4_O_2_, 98%, Sigma-Aldrich) was used as a redox mediator in the electrodeposition process. Nitric acid (HNO₃, 65%, Merck) and sodium hydroxide (NaOH, 99%, Sigma-Aldrich) were used to adjust the pH during deposition. Ethanol (C_2_H_5_OH, 99.9%, Merck) and deionized (DI) water were used for cleaning and dilution, respectively. Fluorine-doped tin oxide (FTO) glass substrates (7 Ω·sq^−1^, Pilkington) served as the conductive support for electrodeposition.

### Fabrication of BiVO_4_ photoanodes

The BiVO_4_ film was deposited using electrochemical deposition. A solution of 0.2962 g Bi(NO_3_)_3_ dissolved in 50 mL distilled water was ultrasonicated for 30 min. Subsequently, 400 mM KI and 5% HNO_3_ were added to adjust the pH to 2. Additionally, 50 mM *p*-benzoquinone (0.2 g) was dissolved in 10 mL of ethanol via ultrasonication for 30 min and added to the solution. The FTO glass substrates were cleaned with ethanol and distilled water via sequential ultrasonication. The BiOI film was electrochemically deposited onto the FTO substrate at various current deposition intensities (14, 22, and 32 mA) using a reference electrode saturated with Ag/AgCl and platinum foil at various potentials vs Ag/AgCl. This process was adapted from the *p*-benzoquinone-based method reported by McDonald and Choi [19], in which benzoquinone serves as a redox mediator for BiOI formation. However, unlike the original study, which applied constant potential conditions, our method employs a variable current-controlled deposition strategy. This approach enables the fine-tuning of the nucleation and growth behavior of BiOI flakes, resulting in enhanced control over the thickness, grain structure, and uniformity, which are key factors that influence the subsequent BiVO_4_ conversion and PEC performance. Then, a 0.2 M VO(acac)_2_ solution in ethanol was coated onto the BiOI film via spin coating with two different volumes of solution (0.4 µL and 0.6 µL). The BiOI film (1 cm × 1 cm) with the VO(acac)_2_ layer was annealed at 450 °C for 2 h. Finally, the BiVO_4_ electrode was rinsed with 1 M NaOH to remove excess V_2_O_5_ from the surface, followed by rinsing with distilled water and drying at room temperature. The photoanode BiVO_4_ was named BiVO_4_(*xy*), where *x* indicates the current intensity for BiOI deposition, and *y* denotes the vanadium precursor volume (*x* = 14, 22, 32; *y* = 4, 6).

### Note on BiVO_4_(144) sample exclusion

The BiVO_4_(144) sample was excluded from the detailed photoelectrochemical (PEC) and comparative analyses because of its poor film uniformity and significantly lower performance metrics. Preliminary characterizations showed that the film exhibited inhomogeneous coverage and an inconsistent PEC response, which could lead to misleading interpretations when comparing the material trends. Therefore, these samples were not included in subsequent analyses to maintain the clarity and consistency of the dataset.

### Characteristics of materials

X-ray diffraction (XRD, Bruker D8 Advance) and Raman spectroscopy (LabRAM Odyssey Semiconductor) were used to analyze the crystal structures of photoanodes. UV–vis absorption spectra were obtained using a Cary 60 spectrophotometer. X-ray photoelectron spectroscopy (XPS, VG ESCALAB250) was employed to determine the chemical states of each photoanode. All photoanode morphologies were examined using field-emission transmission electron microscopy (FESEM, Hitachi SU8010).

### Photoelectrochemical measurements

PEC experiments were performed in a conventional three-electrode cell using an electrochemical workstation (CHI650E, CH Instruments, USA). The three electrodes included a working electrode (BiVO_4_ photoelectrode, 1.0 × 1.0 cm^2^), counter electrode (Pt plate), and reference electrode (Ag/AgCl). The electrolyte used in the photoelectrochemical measurements was 0.50 M Na_2_SO_4_ (pH 5.6), and the xenon lamp was 300 W (PLS-SXE 300C, 100 mW·cm^−2^) equipped with an AM1.5G filter to simulate solar light conditions. Linear sweep voltammetry (LSV) measurements were performed by scanning the potential from −0.6 to 1.2 V (vs Ag/AgCl), at a scan rate of 0.05 V·s^−1^. In the LSV test, the light source illuminated the sample from the back of the FTO glass. Under AM1.5G illumination, electrochemical impedance spectroscopy (EIS) measurements were performed at an open-circuit voltage, covering a frequency spectrum from 1 Hz to 10 kHz. Mott–Schottky curves were recorded at a frequency of 1 kHz in a dark light.

### Applied bias photo to current efficiency

The applied bias photon-to-current efficiencies (ABPEs) of the different photoanodes were determined using [20]:


[1] $$\text{ABPE}\left(\%\right)=100\times \frac{J_{\text{p}}\times \left(E_{\text{rev}}^{\text{ o}}-E_{\text{app}}\right)}{I_{\text{o}}},$$

where *J*_p_ is the photocurrent density (mA·cm^−2^) obtained from the LSV curve, *I*_o_ is the incident light intensity of the solar simulator (100 mW·cm^−2^), and 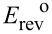 is the standard reversible potential for the water-splitting reaction (1.23 V).

### Incident photon-to-current conversion efficiency

From the excitation at wavelengths of 300–900 nm at 0.5 V, the incident photon-to-current conversion efficiency (IPCE) was evaluated using a chopped monochromator with a 150 W Xe lamp as the simulated light source (developed by HS Technologies, Korea).


[2] $$\text{IPCE}\left(\%\right)=\frac{J\times 1240}{\lambda \times P_{\text{light}}}\times 100\%,$$

where *P*_light_ is the power density of monochromatic light acquired at a given wavelength, and *J* is the photocurrent density (mA·cm^−2^) under illumination at a wavelength (mW·cm^−2^).

## Results and Discussion

### Structural analysis (XRD)

**Figure 1:**
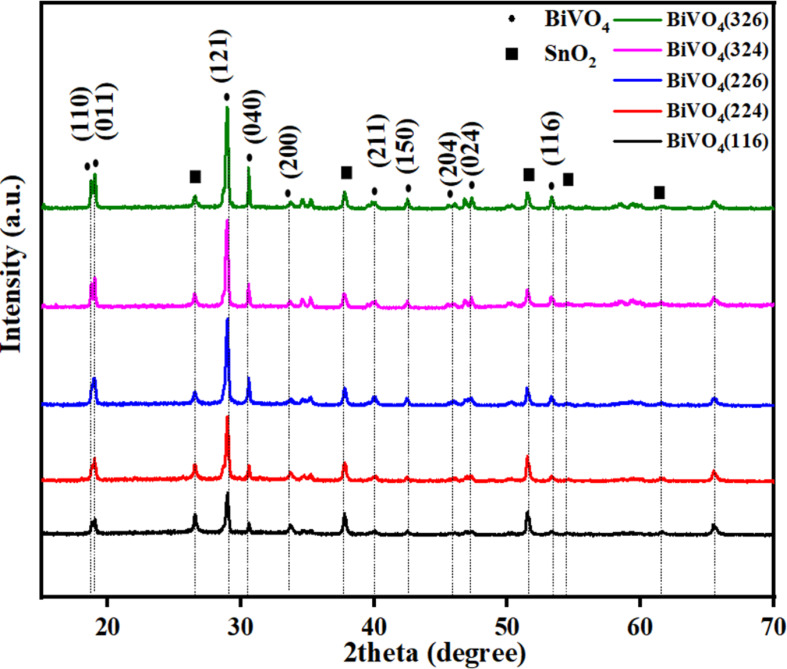
(a) XRD patterns of BiVO_4_(146), BiVO_4_(224), BiVO_4_(226), BiVO_4_(324), and BiVO_4_(326) photoanodes.

X-ray diffraction (XRD) measurements were conducted to investigate the crystal structures of the BiVO_4_ photoanodes under various deposition conditions (BiVO_4_(146), BiVO_4_(224), BiVO_4_(226), BiVO_4_(324), and BiVO_4_(326)), as shown Figure 1. The diffraction peaks of all photoanodes matched those of monoclinic BiVO_4_ (JCPDS PDF #14-0688) and fluorine-doped tin oxide (FTO) substrate (JCPDS PDF #46-1088) [21–23]. The peaks at approximately 28.9°, 30.6°, 34.6°, and 35.2° were assigned to the (110), (121), (040), (200), and (002) planes of monoclinic BiVO_4_, respectively. Notably, the (121) plane exhibited the highest intensity across all samples, which aligns with its high refractive index and superior photocatalytic properties owing to the enhanced adsorption and deionization of water molecules in the structure. Additionally, the (040) peak intensity exhibited a systematic increase at higher electrodeposition current densities, suggesting preferential growth along the crystallographic direction. Higher deposition currents influence ion migration rates and nucleation kinetics, potentially leading to a preferential orientation along the (040) plane. The intensity and sharpness of the peaks increased with increasing current density and VO(acac)_2_ concentration, indicating improved crystallinity and potentially larger grain sizes of the films. This variation in crystallographic properties is expected to influence PEC performance by affecting charge transport and surface reaction kinetics. The average crystallite size (grain size) of the BiVO_4_ films was estimated using the Scherrer equation based on the full width at half maximum (FWHM) of the (121) diffraction peak.

The Scherrer equation to calculate average crystallite size (grain size) [24–25] is


[3] $$D=\frac{k\lambda }{\beta \cos\theta },$$

where λ is the X-ray wavelength, θ is the Bragg angle in radians, β is the full width at half maximum of the peak in radians, *D* is the particle size, and *k* is a constant with a value of 0.9.

As shown in Table 1, an increase in the deposition current and VO(acac)_2_ concentration led to narrower peak widths and larger crystallite sizes, indicating an improved crystalline quality. BiVO_4_(326) exhibited the largest average crystallite size (≈40 nm), consistent with the enhanced PEC performance and reduced lattice strain observed in the Raman analysis.

**Table 1:** Estimated crystallite sizes of BiVO_4_ photoanodes (from the Scherrer equation).

Sample	2θ– (121) Peak	FWHM (β) (°)	Crystallite size *D* (nm)	Estimated particle size (FESEM) (nm)	Notes

BiVO_4_(146)	28.76	0.32	25.1	**≈**400–500	large, irregular particles
BiVO_4_(224)	28.82	0.29	27.6	**≈**300–400	rougher morphology
BiVO_4_(226)	28.88	0.26	30.8	**≈**250–350	more uniform particles
BiVO_4_ (324)	28.94	0.23	34.9	**≈**300–450	larger, loosely packed
BiVO_4_(326)	28.99	0.20	40.1	**≈**200–300	densely packed, porous

### Morphological characterization (FESEM)

**Figure 2:**
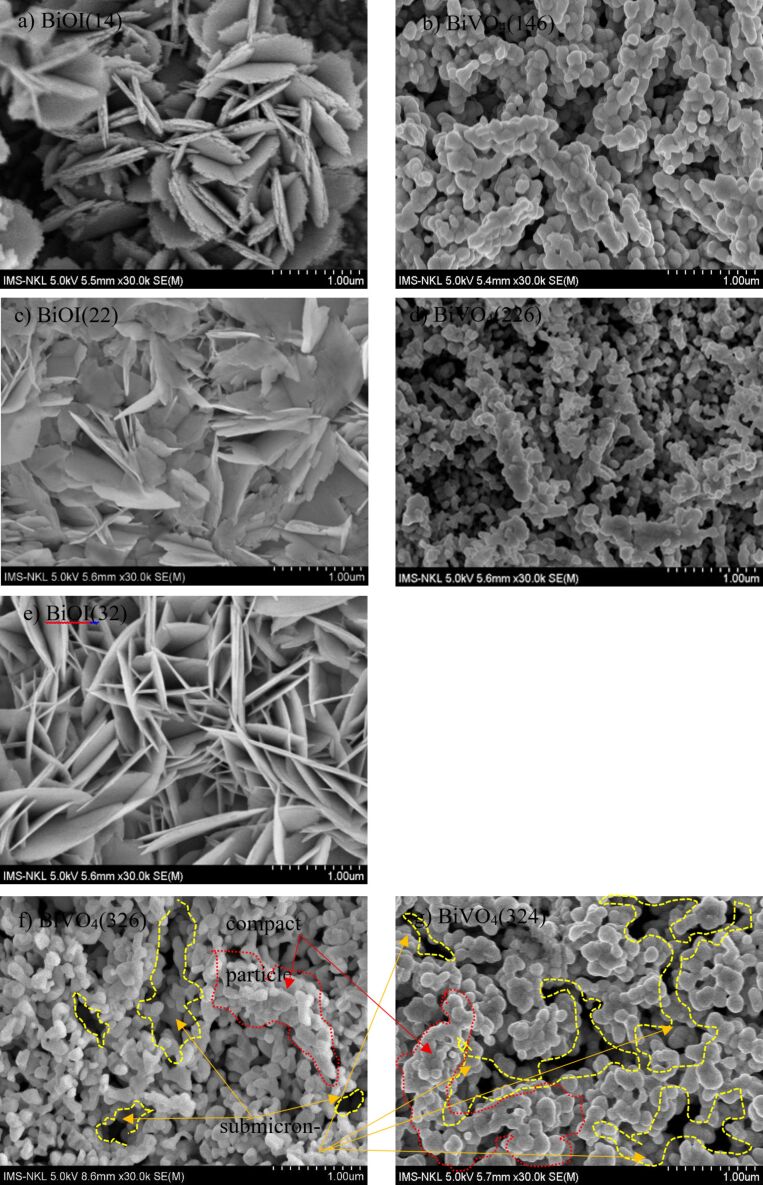
FESEM images of (a), (c), and (e) BiOI film under various intensities of current deposition, and (b) BiVO_4_(146), (d) BiVO_4_(226), (f) BiVO_4_(326), and (g) BiVO_4_(324) films.

Field-emission scanning electron microscopy (FESEM) images highlighted the evolution of the surface morphology under different fabrication conditions. The transition from two-dimensional plate-like BiOI crystals to three-dimensional BiVO_4_ particles was accompanied by the formation of submicrometer-scale voids, indicative of grain growth and recrystallization during the annealing process (Figure 2a,c,e). At lower current densities (e.g., BiVO_4_(146)), the BiVO_4_ films exhibited larger and more irregular particles with relatively low surface coverage (Figure 2b). In contrast, higher current densities (e.g., BiVO_4_(324) and BiVO_4_(326)) resulted in more uniform and closely packed particles, along with visible submicrometer-scale voids (Figure 2f,g). This morphology maximizes the active surface area available for photoelectrochemical reactions. Additionally, the observed submicrometer voids, which were more prominent in films deposited with larger VO(acac)_2_ volumes, suggested improved charge separation and transport pathways. These voids facilitate the diffusion of the reactants and products, reducing the recombination rates and enhancing the water-splitting efficiency. The finer particle sizes and increased porosity, as observed for BiVO_4_(326), align with the optical and Raman results, highlighting the impact of the optimized deposition parameters on PEC performance. The FESEM images also revealed that the films prepared at higher current densities exhibited well-defined grain boundaries, which correlated with the reduced lattice strain observed in the Raman spectra. These structural features are critical for improving the electronic and catalytic properties of BiVO_4_ photoanodes.

To complement the XRD-derived crystallite sizes, the particle sizes were estimated from the FESEM images. As shown in Table 1, the particle size trends do not perfectly follow the crystallite size evolution. For instance, BiVO_4_(326) exhibits the largest crystallite size (≈40 nm) but has smaller surface particle aggregates (200–300 nm) than BiVO_4_(324). This mismatch arises because the particles observed via SEM are often composed of multiple crystalline grains. This distinction suggests that while the crystallite size governs the internal crystalline quality and carrier mobility, the particle size affects the surface area and interface kinetics. Ideally, a material such as BiVO_4_(326) with both large crystallites and small, porous particles offers superior PEC performance owing to improved charge transport and enhanced surface reaction sites.

### Optical properties (UV–vis)

**Figure 3:**
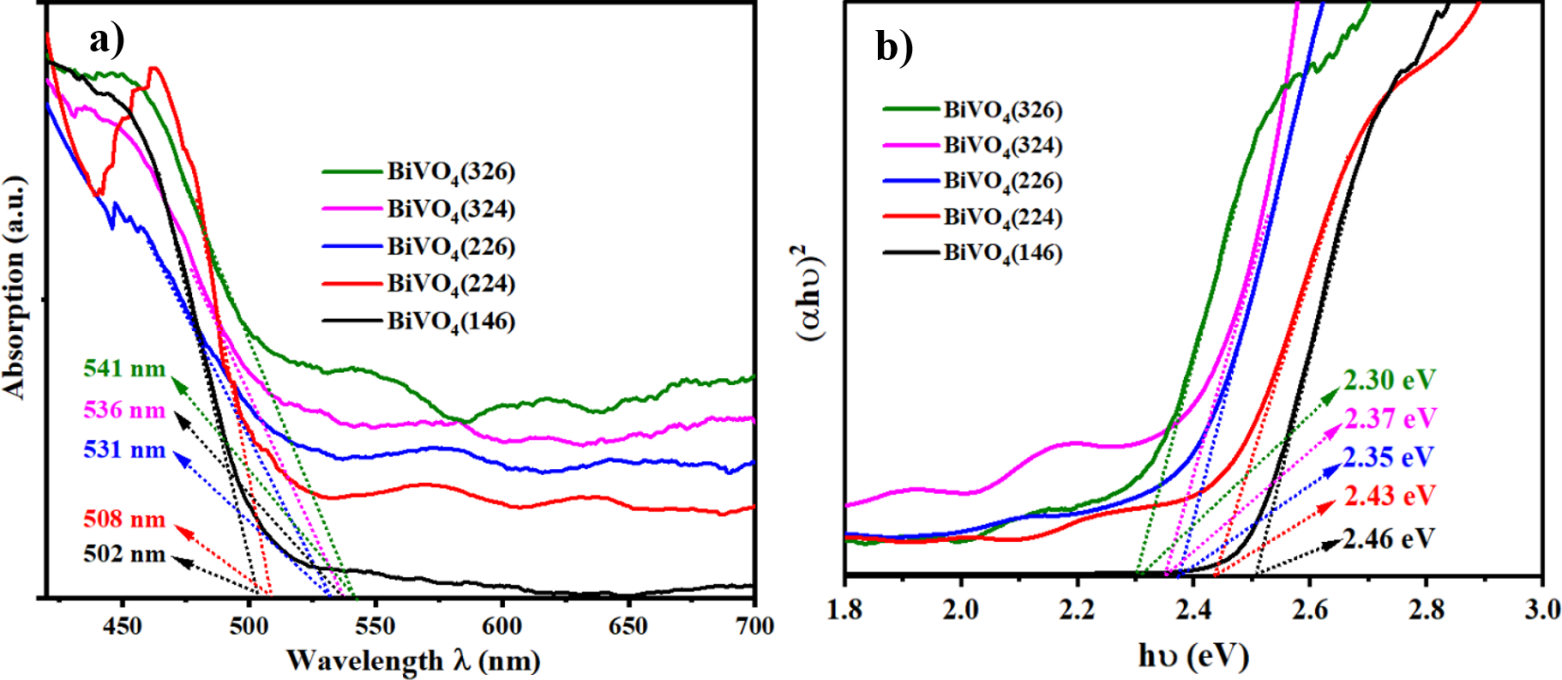
(a) UV–vis spectrum, (b) bandgap energies of BiVO_4_(146), BiVO_4_(224), BiVO_4_(226), BiVO_4_(324), and BiVO_4_(326) photoanodes. Minor fluctuations above 500 nm are due to light scattering from porous films.

UV–vis absorption spectroscopy (Figure 3) showed that BiVO_4_ samples absorb visible light, with absorption edges between 502 and 541 nm and optical bandgaps between 2.46 and 2.30 eV (Figure 3b). Bandgap values were determined using Tauc plots for indirect allowed transitions, based on (α*h*ν)^2^ ∝ (*h*ν – *E*_g_), where α is the absorption coefficient, *h* is Planck’s constant, ν is the frequency, and *E*_g_ is the bandgap energy. The (α*h*ν)^2^ values plotted against the photon energy determined *E*_g_ at the absorption edge intersection, as shown Figure 3b. Samples prepared with higher electrodeposition currents and larger VO(acac)_2_ amounts exhibited redshifted absorption edges, indicating enhanced light harvesting due to improved crystallinity and reduced disorder. XRD and Raman spectroscopy confirmed these improvements through stronger peaks, suggesting fewer defects. The decrease in the bandgap (≈0.16 eV) is consistent with research linking oxygen vacancies to band tailing in BiVO_4_ films [26]. Besides, Figure 3a shows that the BiVO_4_(326) and BiVO_4_(324) samples have absorption that goes beyond 520 nm, with some absorption still measurable up to about 650 nm. This sub-bandgap absorption occurs because of the creation of mid-gap states, mainly caused by missing oxygen atoms and structural issues that arise during high-current electrodeposition or when using higher amounts of the VO(acac)_2_ precursor. The redshifted tails indicate that there are special energy states in the material that allow it to absorb light even at energies lower than those normally expected. The BiVO_4_(326) sample, in particular, exhibited the most pronounced tailing, consistent with its enhanced photoelectrochemical performance. The evidence suggests an optimal concentration of oxygen vacancies that broadens light absorption while avoiding excessive recombination of the charge carriers. In contrast, BiVO_4_(146) has a clear absorption edge and very little tailing, indicating that it has fewer defects but does not absorb light well beyond 520 nm. These findings match other studies that connect oxygen vacancies to the spread of light absorption and smaller optical bandgaps in BiVO_4_ [26].

### Vibrational properties (Raman)

**Figure 4:**
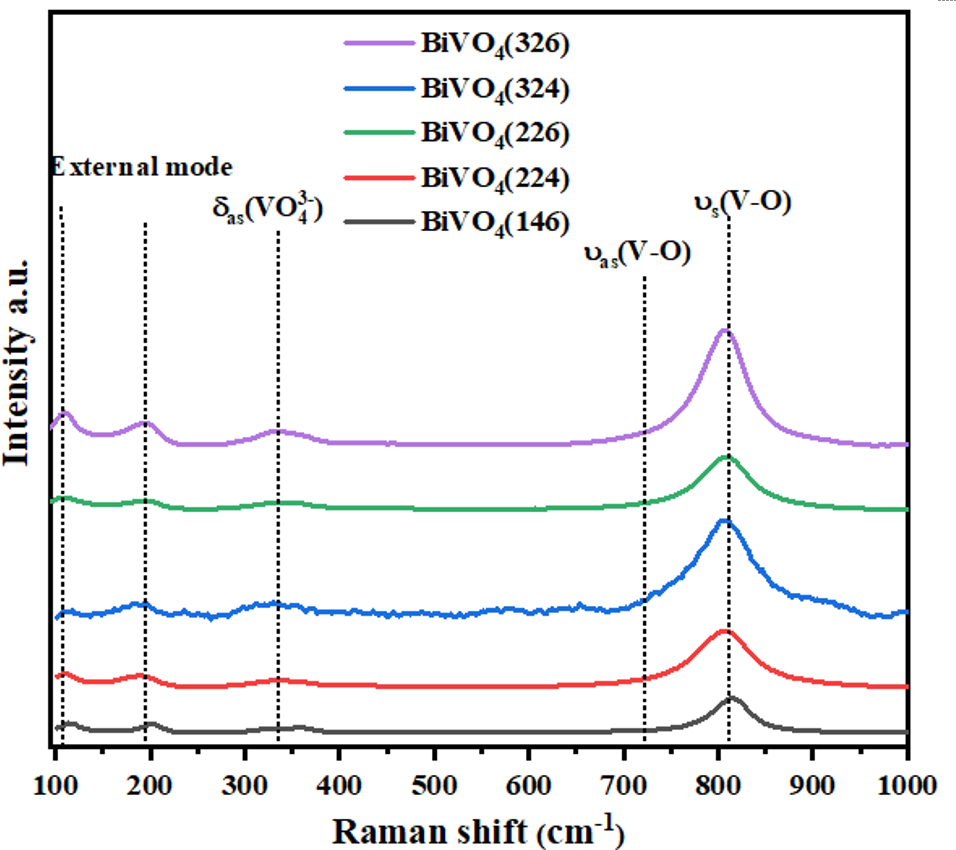
Raman spectrum of BiVO_4_(146), BiVO_4_(224), BiVO_4_(226), BiVO_4_(324), and BiVO_4_(326) photoanodes.

The Raman spectra (Figure 4) corroborated the XRD findings, displaying characteristic peaks of monoclinic BiVO_4_ at 219, 329, 370, 712, and 830 cm^−1^ [27]. These bands correspond to the vibrational modes associated with the VO_4_^−^ tetrahedral structure, which directly affects the electronic properties of the material [28]. The band at 830 cm^−1^, assigned to the symmetric stretching of the V–O bond [29], appears in samples prepared at higher current densities and larger VO(acac)₂ volumes. This observation suggests that improved crystallinity enhances the structural uniformity of the VO_4_^−^ tetrahedra, leading to more efficient charge-transfer pathways. Furthermore, the appearance of sharper and more intense Raman peaks with increasing deposition parameters indicates a reduction in the structural defects and lattice strain. The vibrational modes at 329 cm^−1^ and 370 cm^−1^, corresponding to the asymmetric and symmetric deformations of the V–O bond [30–31], respectively, showed a strong correlation with XRD-derived grain size variations. Larger grains typically result in fewer grain boundaries, reducing phonon scattering and enhancing vibrational coherence. Raman spectra also provide insights into the influence of fabrication conditions on surface chemistry. The relative intensities of the peaks suggest that higher deposition currents promote the formation of active crystal facets, which are critical for PEC performance. These results align with the enhanced photoelectrochemical activity observed for BiVO_4_(326), as the improved vibrational characteristics reflect more efficient light absorption and charge separation.

### Photoelectrochemical performance

**Figure 5:**
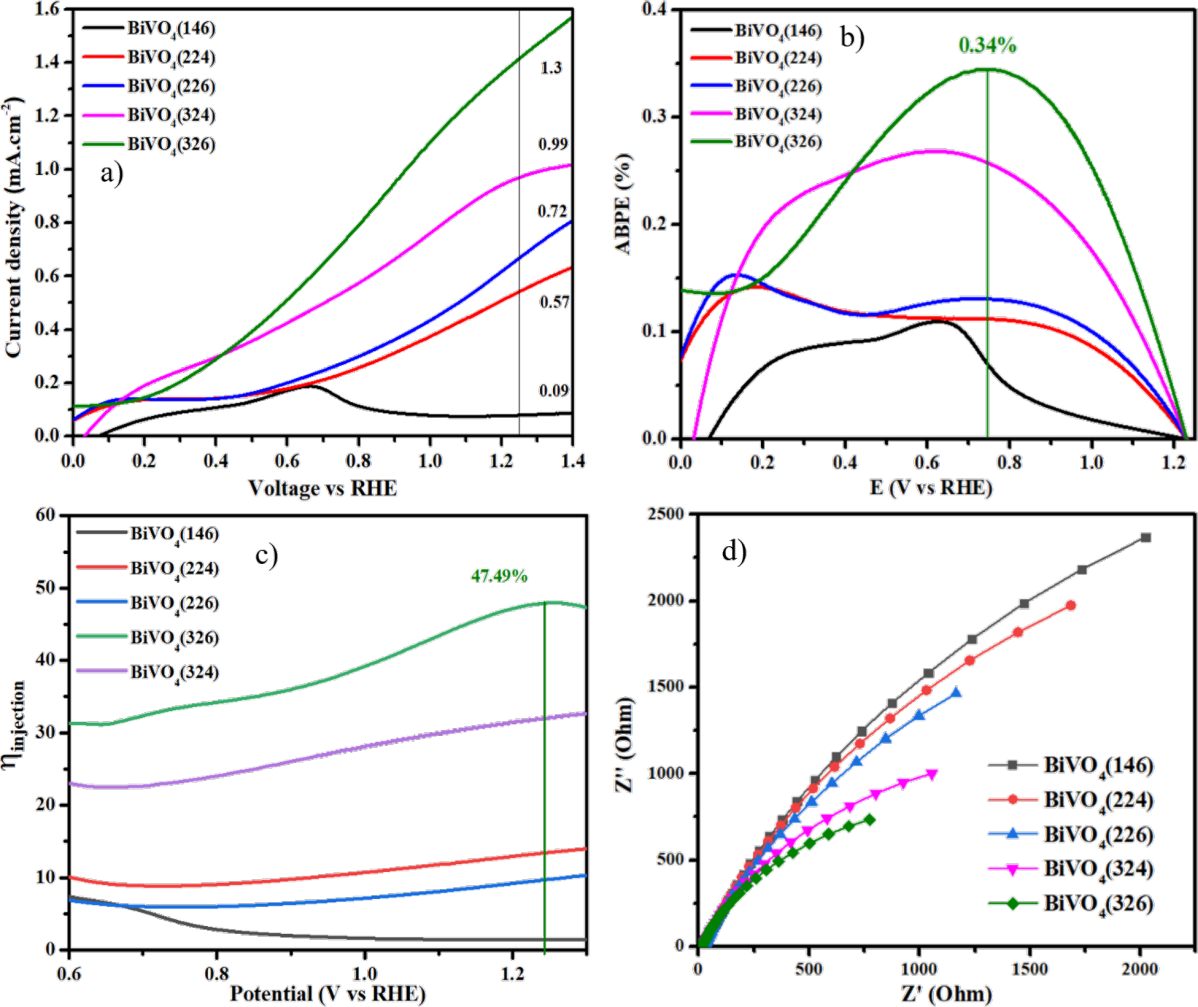
(a) LSV curves, (b) ABPE curves, (c) hole injection efficiency (η_surface_) curves, and (d) EIS plots for each photoanode.

Linear sweep voltammetry (LSV) was used to evaluate the water-splitting efficiency of the photoelectrochemical (PEC) system in a 0.50 M Na₂SO_4_ solution (pH 5.6) under AM1.5G illumination (100 mW·cm^−2^). The optimal PEC performance was achieved for the BiVO_4_(326) sample, synthesized using a current intensity of 32 mA and VO(acac)_2_ precursor volume of 0.6 µL. As shown in Figure 5a, this sample exhibited the highest photocurrent density of 1.2 mA·cm^−2^ at 1.23 V vs RHE, indicating efficient photoelectrochemical activity. This superior performance can be attributed to the combined effects of enhanced crystallinity, film morphology, and preferential crystal orientation. As shown in the XRD patterns (Figure 1), BiVO_4_(326) displays the most intense (121) and (040) diffraction peaks among all the samples, suggesting preferred growth along these planes, which are known to facilitate efficient charge separation and transport. Previous studies have reported that the (121), (040), and (010) facets of monoclinic BiVO_4_ contribute to enhanced photocatalytic activity by serving as active sites for oxidation and reduction reactions [32–33]. In particular, the large surface area of the (010) facet is associated with the effective suppression of charge recombination. Although other samples also exhibited these orientations, the relatively higher (121)/(040) intensity ratio and sharper peaks for BiVO_4_(326) indicate improved structural ordering, which, together with its more porous and interconnected morphology (Figure 2), likely promotes better carrier mobility and PEC efficiency.

The ABPE curves (Figure 5b) demonstrate that BiVO_4_(326) achieved the highest efficiency of 0.4% at 0.8 V vs RHE, compared to the lower efficiencies of the other samples. This improvement is attributed to the synergistic effects of enhanced light absorption, reduced charge recombination, and increased charge transport efficiency, which are facilitated by the optimized fabrication parameters.

To investigate the influence of different chemical components on charge separation within the BiVO_4_ bulk and surface charge transfer, LSV experiments were conducted in a 0.50 M Na_2_SO_4_ (pH 5.6) solution with 1 M Na_2_SO_3_ as a hole scavenger (Figure 5c). The hole injection efficiency was calculated as the ratio of the photocurrent density values obtained with and without the Na_2_SO_3_ hole scavenger. The hole injection efficiency of the BiVO_4_(326) photoanode at 1.23 V vs RHE was 47%, which was higher than that under other conditions. This indicates that the formation of films with larger surface areas reduces the charge recombination rate and facilitates faster charge transfer rates.

AC impedance measurements were performed on the photoanodes to assess their charge transfer capabilities. As shown in Figure 5d, BiVO_4_(326) exhibited a smaller impedance arc under illumination, indicating a minimal charge-transfer resistance. This is favorable for the rapid utilization of photogenerated holes in water oxidation reactions. Overall, these results demonstrate that the optimized BiVO_4_(326) configuration provides improved photocatalytic activity by enhancing charge transport, reducing recombination, and maximizing the active surface area for efficient PEC water splitting.

**Figure 6:**
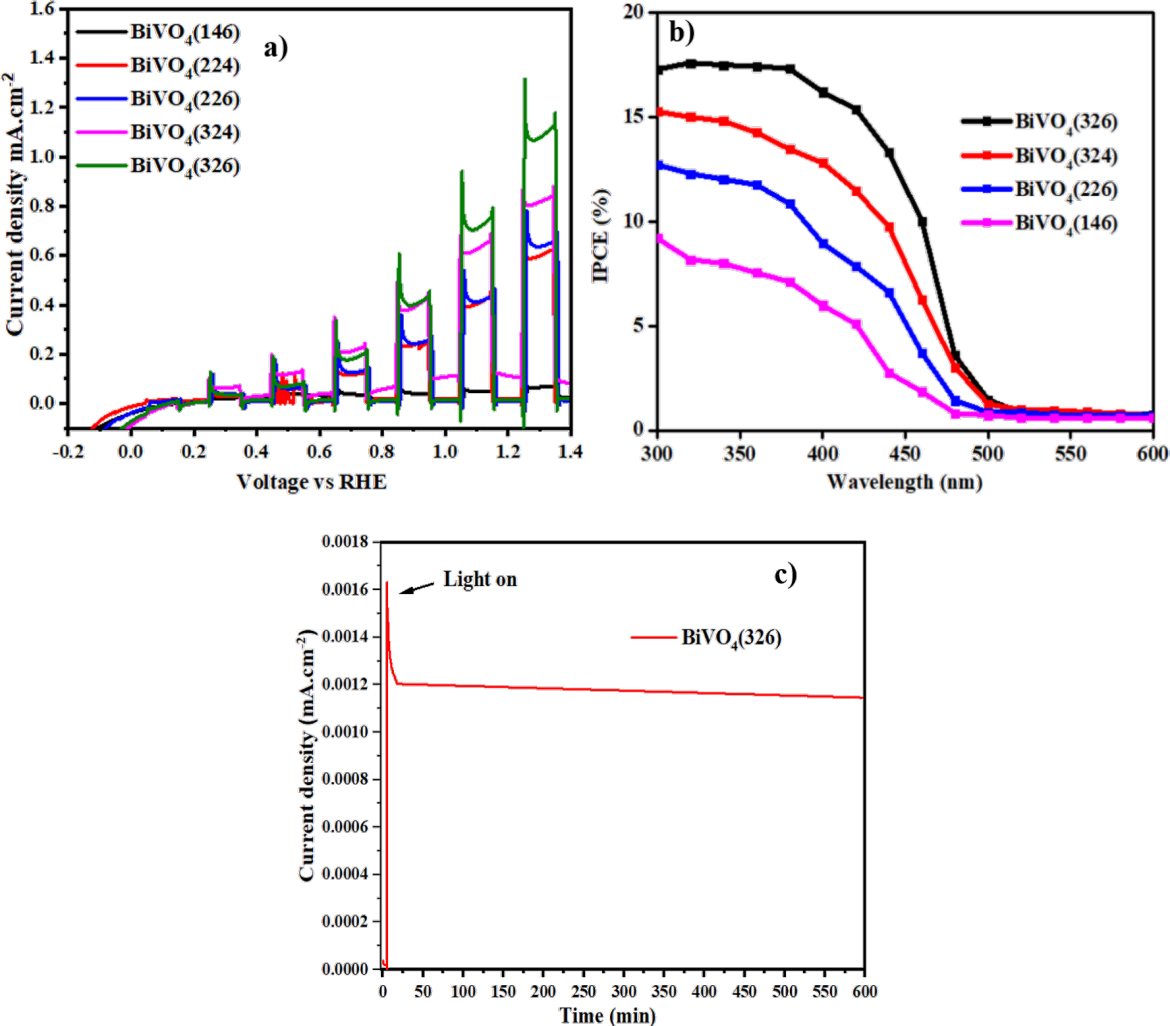
(a) LSV curves under chopped illumination, (b) IPCE curves for each photoanode, and (c) chronoamperometry curve of BiVO_4_(326) measured at 1.23 V vs RHE under continuous AM1.5G illumination in 0.50 M Na_2_SO_4_ for 600 min.

The LSV results revealed that the photoanodes fabricated at higher current densities and larger VO(acac)_2_ volumes (e.g., BiVO_4_(326)) exhibited the highest photocurrent density at 1.23 V vs RHE, reaching 1.2 mA·cm^−2^. This is nearly 4.9 times higher than that of BiVO_4_(146). The enhanced photocurrent density correlated with the increased crystallinity, optimized grain size, and improved surface area observed in the XRD, Raman, and FESEM analyses, which collectively improved charge separation and transport. The chopped illumination data (Figure 6a) confirmed stable photoresponses with minimal decay over time, indicating the high photostability of the optimized photoanodes.

The IPCE measurements (Figure 6b) over the wavelength range of 300–500 nm showed that BiVO_4_(326) exhibited a peak IPCE value of 18.1%, which was significantly higher than the 8.9% of BiVO_4_(146). This increase is consistent with the improved optical absorption and structural properties, as well as the reduced electron–hole recombination rates observed in the Raman and UV–vis analyses.

The long-term photoelectrochemical stability of the optimized BiVO_4_(326) photoanode was evaluated using chronoamperometry under continuous AM1.5G illumination at 1.23 V vs RHE in 0.50 M Na_2_SO_4_ electrolyte (Figure 6c). The photocurrent density initially peaked at approximately 1.6 mA·cm^−2^ and stabilized quickly at ≈1.2 mA·cm^−2^, maintaining this value consistently for 600 min (10 h) of operation. This stable behavior demonstrates the excellent PEC durability of the BiVO_4_(326) photoanode and confirms the robustness of its structural and surface properties under prolonged operational conditions. The stability is attributed to the optimized crystallinity, reduced recombination, and uniform morphology achieved through controlled-intensity current electrodeposition.

The PEC performance of BiVO_4_(326) was benchmarked against similar studies employing hydrothermal synthesis, direct electrodeposition, and BiOI-derived conversion methods. As summarized in Table 2, our sample achieved a photocurrent density of 1.2 mA·cm^−2^ at 1.23 V vs RHE, which is comparable to or better than the values reported for other BiVO_4_-based photoanodes. Notably, this performance was achieved under mild synthesis conditions and was supported by excellent long-term stability (600 min), which many previous reports do not provide. The enhanced performance in our study can be attributed to the optimized grain structure, controlled crystallinity, and superior film uniformity achieved via the controlled-intensity current electrodeposition of BiOI.

**Table 2:** Summary of recent single-layer BiVO_4_ photoanodes and their PEC performance.

Study	Method	Photocurrent density (mA/cm^2^)	Stability (duration)	Notes	Ref

McDonald and Choi, (2012)	BiOI-derived BiVO_4_ (simple electrodeposition)	≈1.25 @ 0.5 V	not reported	BiOI precursor, no stability test	[19]
Kim et al. (2014)	electrodeposition of BiVO_4_	≈1.4 @ 1.23 V	not reported	nanoporous BiVO_4_	[18]
Yun He et al. (2017)	hydrothermal BiVO_4_	≈0.81 @ 1.23 V	few minutes	flower-like BiVO_4_, high recombination	[15]
Mohamed et al. (2021)	electrodeposition (needle-like nanoflower)	≈0.32	not reported	petal-like morphology, 7 min ED time	[21]
Fuentes-Camargo et al. (2020)	pulse plating of Bi, then conversion to BiVO_4_	≈0.35	stable and reusable	focused on pollutant degradation under Xe lamp	[34]
Pelissari et al. (2021)	SILAR (5 cycles), annealed	1.95	not reported	highly optimized multilayer thin film	[29]
Qiuhang Lu et al. (2022)	RF magnetron sputtering (BiVO_4_ target)	≈2.1	not reported	precise thickness control, but lower crystallinity	[35]
this work	controlled-intensity BiOI → BiVO_4_ (electrodeposition)	1.2	600 min stable	tuned crystallinity, porosity, and facet orientation	

### Surface chemical composition (XPS)

**Figure 7:**
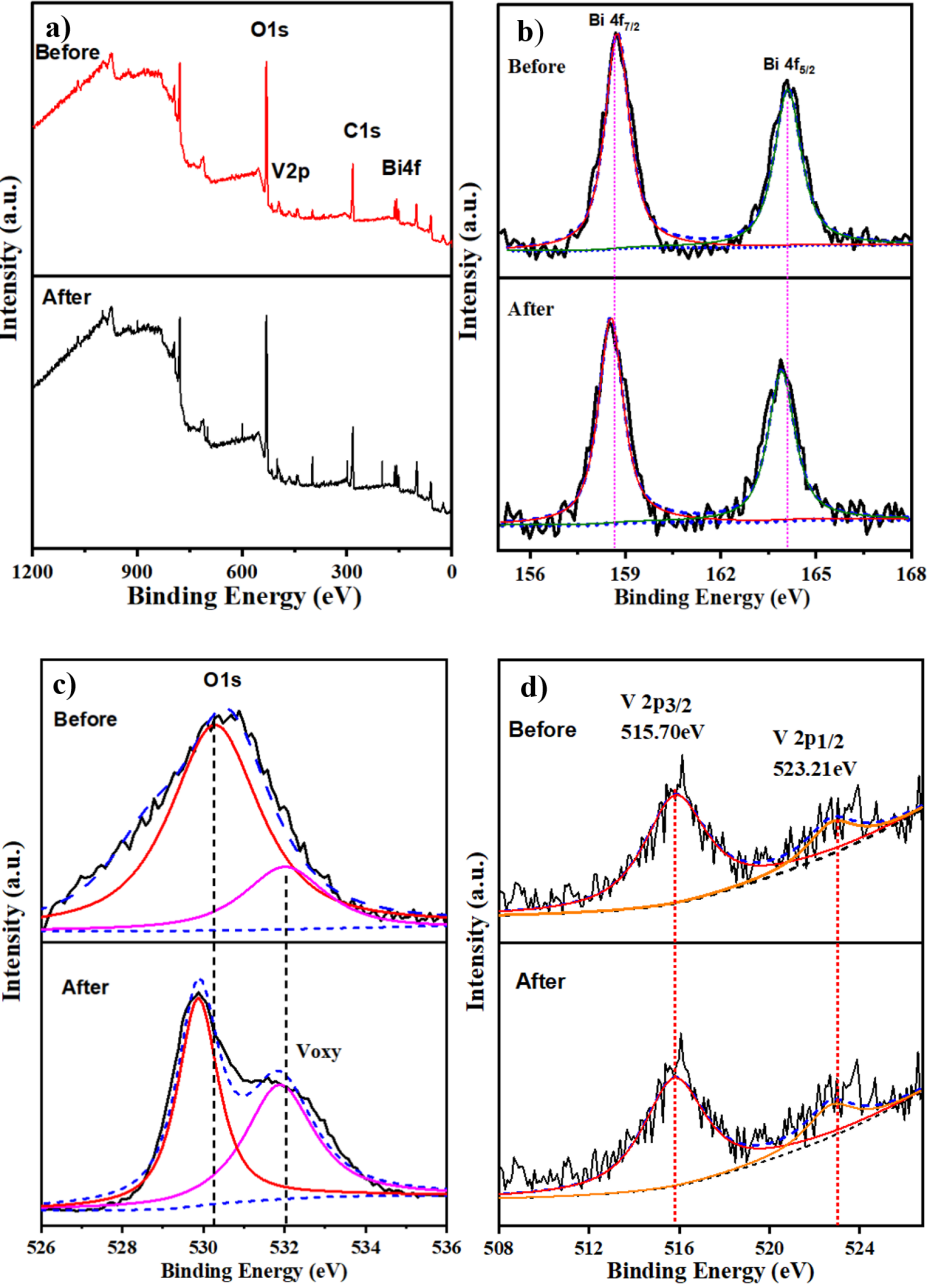
X-ray photoelectron spectroscopy (XPS) analysis of the BiVO_4_(326) photoanode before and after PEC stability testing in 30 min (0.5 M Na_2_SO_4_). (a) XPS view scans of survey spectrum and high-resolution spectra of (b) Bi 4f, (c) O 1s, and (d) V 2p.

X-ray photoelectron spectroscopy (XPS) was employed to investigate the surface chemical states and composition of the BiVO_4_ photoanodes, focusing on the optimized BiVO_4_(326) sample. The survey spectrum (Figure 7a) confirmed the presence of Bi, V, and O, which is consistent with the BiVO_4_ structure. High-resolution Bi 4f spectra (Figure 7b) show a characteristic doublet at binding energies of 159.0 eV (Bi 4f_7/2_) and 164.2 eV (Bi 4f_5/2_), corresponding to Bi^3+^ in the monoclinic phase of BiVO_4_ [36–37]. The V 2p region (Figure 7d) exhibits peaks at 516.2 eV (V 2p_3/2_) and 524.1 eV (V 2p_1/2_), indicating the presence of V^5+^ species associated with the VO_4_^3−^ tetrahedra [38–39]. The Bi 4f and V 2p spectra remained unchanged after testing, confirming chemical and structural stability of the electrode during long-term PEC operation. The O 1s spectrum (Figure 7c) was deconvoluted into two main peaks: the dominant peak at 530.1 eV is attributed to lattice oxygen (O^2−^), while the broader shoulder around 531.8 eV corresponds to surface hydroxy groups and oxygen vacancies [40–42]. Notably, post-PEC testing revealed an increase in the intensity of the oxygen vacancy-related component, suggesting the formation or activation of surface defects during prolonged photoelectrochemical operation. These oxygen vacancies are known to play a crucial role in enhancing charge separation and facilitating surface water oxidation reactions, thereby contributing to improved catalytic performance [43]. The post-stability XPS analysis also showed that BiVO_4_(326) was chemically stable and that oxygen vacancies helped maintain its long-term photoelectrochemical activity.

## Conclusion

This study demonstrates the successful synthesis of high-performance BiVO_4_ photoanodes through controlled-intensity current electrodeposition, emphasizing the critical role of fabrication conditions on the structural, optical, and photoelectrochemical properties. XRD and Raman analyses confirmed the enhanced crystallinity and reduced lattice strain in the samples prepared under higher current densities and greater VO(acac)_2_ volumes, which correlated with improved charge transport and reduced recombination losses. UV–vis absorption spectroscopy and FESEM imaging revealed that the optimized conditions led to better light-harvesting capabilities and enhanced surface area owing to finer particle morphologies and increased porosity of the photocatalysts. XPS analysis highlighted the presence of oxygen vacancies and well-defined chemical states, further contributing to the improved catalytic activity and charge separation. Photochemical measurements demonstrated that the BiVO_4_(326) sample achieved the highest photocurrent density of 1.2 mA·cm^−2^ at 1.23 V vs RHE, a surface hole injection efficiency of 47%, and a peak IPCE of 18.1%, outperforming the other samples. These results highlight the synergistic effects of an improved crystalline structure, optimized morphology, and enhanced electronic properties. This study provides a comprehensive understanding of the interplay between fabrication parameters and PEC performance, paving the way for efficient BiVO-based photoanodes for solar-driven water-splitting applications.

## Data Availability

Data generated and analyzed during this study is available from the corresponding author upon reasonable request.
